# The Potential Role of Korean Mistletoe Extract as an Anti-Inflammatory Supplementation

**DOI:** 10.1155/2021/2183427

**Published:** 2021-06-29

**Authors:** Soo-Min Ha, Ji-Hyeon Kim, Jong-Won Kim, Do-Yeon Kim, Min-Seong Ha

**Affiliations:** ^1^Laboratory of Exercise Physiology, Department of Physical Education, Pusan National University, 2 Busandaehak-ro 63beon-gil, Geumjeong-gu, Busan 46241, Republic of Korea; ^2^Department of Liberal Arts, Mokpo National Maritime University, 91 Haeyangdaehak-ro, Mokpo-si, Jeollanam-do 58628, Republic of Korea; ^3^Department of Physical Education, Busan National University of Education, 33 Gyodae-ro, Yeonje-gu, Busan 47503, Republic of Korea; ^4^Department of Sports Culture, College of the Arts, Dongguk University-Seoul, 30 Pildong-ro 1-gil, Jung-gu, Seoul 04620, Republic of Korea

## Abstract

Korean *mistletoe* has anti-inflammatory and antioxidant functions and may be a useful training supplement. We investigated the effect of Korean *mistletoe* extract (KME) on inflammatory markers after high-intensity exercise by 20 university male rowers (KME group vs. CON group) consuming 110 mL KME/dose (2 times a day over 8 weeks). Blood samples were collected for measurement of serum cytokine levels at baseline, immediately after exercise, and following 30 minutes of recovery. Interleukin-6 (IL-6), tumor necrosis factor-alpha (TNF-*α*), and C-reactive protein (CRP) were used as markers for inflammation. After supplementation, IL-6 and TNF-*α* levels were significantly lowered in the KME group than in the CON group at baseline, immediately after exercise, and following 30 minutes of recovery. KME can reduce high-strength exercise-induced increases in the levels of serum inflammatory cytokines in active individuals and improve anti-inflammatory functions.

## 1. Introduction

Exercise promotes health, prevents disease, increases life expectancy, and is considered to some to be a medicine. However, elite athletes are a paradox that should be emphasized [1]. For athletes to achieve a higher rank or exceptional results in competitive sport, tolerance for strenuous exercise is necessary.

Strenuous exercise may serve as a physical stressor and can thus create a state similar to an inflammatory response [2]. Inflammation is a tissue's immune response to various stimuli [3], and during inflammatory processes by macrophages and inflammatory cells, inflammatory mediators including nitric oxide, cytokines, and chemokines are released [4]. As such, arduous conditions during exercise activate neutrophils, monocytes, and macrophages. It can also increase levels of proinflammatory cytokines including interleukin-1 beta (IL-1*β*), tumor necrosis factor-alpha (TNF-*α*), and interleukin-6 (IL-6) and amplify different aspects of inflammation through cytokine action [5]. These factors impede exercise performance and increase interactions between serum inflammatory cytokines and other organ systems, resulting in signs and symptoms of inflammation [1, 6]. TNF-*α* is the major active cytokine during an acute inflammatory response and sends a stimulatory signal to leukocytes at sites of inflammation to eliminate microorganisms and decrease inflammation [7]. Activation of nuclear factor kappa-light-chain-enhancer of activated B cells (NF-*κ*B), in combination with the TNF receptor 1, exerts a negative effect on induction of intracellular gene expression in response to oxidative stress [8]. IL-6 is a cytokine that is essential to the transport of proteins that regulate immunity. IL-6 levels change significantly in response to exercise. It is mostly activated during skeletal muscle contraction and plays crucial roles in the control of immune and metabolic functions [9]. IL-6 simultaneously regulates both inflammatory and anti-inflammatory functions [10, 11]. Inflammatory proteins, enzymes, and C-reactive protein (CRP) have been used as biomarkers in medicine to assess levels of inflammation, infection, and injury [12].

Nutrition planning may be useful to counteract inflammatory responses after strenuous exercise, and dietary supplements are able to inhibit inflammatory response and specific immune functions [13]. Thus, many studies have attempted to establish the importance of nutrition that enhances immunity during athlete training and during competition [14]. Dietary supplements with anti-inflammatory and antioxidant functions may aid athlete recovery after repetitive intense exercise [15]. To characterize the effects of such dietary supplements on exercise physiology, future studies should be planned carefully and with precision. It is important to determine the effect of specific supplements on exercise physiology, as opposed to having performance as the sole consideration [16]. Vegetable polyphenols are known to have various biological effects, and administration of polyphenols can reduce oxidative stress and levels of proinflammatory cytokines [17].

*Mistletoe* (*Viscum album* var. *coloratum*) is an angiosperm that survives by absorbing moisture, minerals, and organic materials from several host trees. As it retains photosynthetic function, this makes it a hemiparasite that is not entirely dependent on its host. *Mistletoe* is chemically composed of viscotoxins, lectins, flavonoids, phenolic acids, terpenoids, sterols, phenylpropanoids, and alkaloids [18]. It has been reported to be effective not only as an antitumor [19] and anti-inflammatory agent [20] but also for immune enhancement and regulation [21] and treatment of atherosclerosis and hypotension [22]. *Mistletoe* also promotes antioxidant functions [23], increases endurance through increased mitochondrial activity [24], and has an antidiabetic effect through insulin secretion [25]. As such, *mistletoe* is used as a supplement to ameliorate symptoms of the above illnesses.

Excessive long-term training and intense exertion during exercise can cause expression of inflammatory cytokines. Various measures have been explored to minimize this response, and dietary supplements with anti-inflammatory and antioxidant effects may help athletes to recover from repetitive intense exercise and thus prevent reductions in performance. To address this, we conducted this study to determine the effects of *mistletoe* extract supplementation on levels of inflammatory markers in university-aged male rowing athletes during an 8-week training period. The performance measure for high-intensity aerobic and anaerobic sport involved rowing a 2,000 m distance on an ergometer.

## 2. Methods

### 2.1. Subjects

Study subjects included 20 university-aged male rowing athletes (means ± SD: age = 20.37 ± 1.20 year, height = 179.96 ± 4.33 cm, weight = 80.57 ± 8.73 kg) at H University, Busan, Republic of Korea. Subjects individually had more than 5 years of rowing experience and were national-level athletes who won at national competitions. This study was conducted in accordance with protocols approved by the institutional review board at Pusan National University (PNU IRB/2016_24_HR). The subjects were fully informed about the study and understood its objective and intent prior to participation. Only those who gave voluntary written consent were included in this study. All subjects were athletes without a prior record of musculoskeletal or other injuries and had not been treated with medication in the 6 months prior to the study. The subjects were prohibited from taking other nutritional supplements or drugs for a month before commencement of the study and for its duration.

### 2.2. Study Design

**Figure 1:**
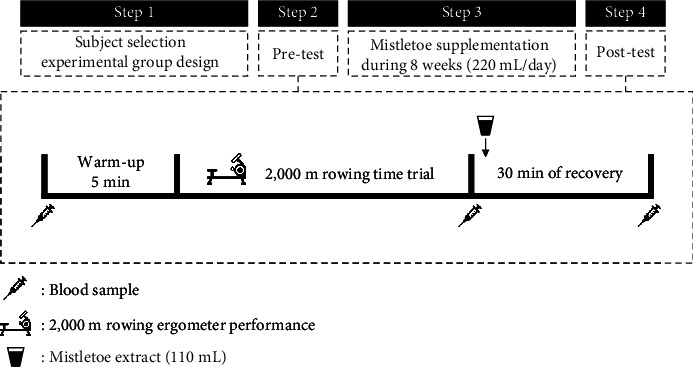
Overview of intervention study design. The pre- and postsupplementation was performed in the experiment in the same condition and methods. Rowing performance measures (2,000 m rowing ergometer race simulation) were completed before and after each supplementation period (the mistletoe supplement group and the control group). Venous blood samples at the baseline were assessed before the subjects underwent rowing ergometer test. After the exercise, venous blood measures were assessed immediately and after 30 minutes. The KME group took 110 mL of mistletoe extract immediately after the exercise.

Subjects were randomly divided into two groups: the experimental group that was administered the Korean *mistletoe* extract (KME) supplement (*n* = 10) and the control (CON) group (*n* = 10). All subjects undertook preseason training for 8 weeks (January to February), and the KME group were administered *mistletoe* extract during this period. All of the following parameters were measured using the same protocol and under the same conditions: 2,000 m rowing ergometer performance was assessed and serum IL-6, TNF-*α*, and CRP levels were measured before (0 week) and after (8 weeks) the training period. They were measured at baseline, immediately after the 2,000 m rowing exercise, and following 30 min of recovery. We set the recovery time of inflammation markers through mistletoe extract intake to 30 minutes based on previous recovery studies after 2,000 m rowing ergometer performance of rowers [26, 27]. The KME group was also administered *mistletoe* extract (110 mL) and had 30 min of recovery after the rowing test. The study design is shown in Figure 1.

### 2.3. Supplementation

The KME group was administered 220 mL of *mistletoe* extract (*mistletoe* juice, Jinheong Food Ltd., Uljin, South Korea) daily during the 8-week training period, divided into two doses. A 110 mL dose was administered once every morning and again 1 h after the evening meal [28]. The KME group was administered an additional 110 mL of *mistletoe* extract before and immediately following the 2,000 m rowing exercise, prior to blood collection. *Mistletoe* extract was prepared according to a method validated in previous studies on natural plants [28, 29]. The specific manufacturing process is as follows. Oak mistletoe (*Viscum album* var. *coloratum*) grown at an altitude of 800 m or higher in Uljin (Mt. Tonggo and Mt. Baekam) in Gyeongsangbuk-do, Republic of Korea, was harvested and dried naturally for 1 month. After that, it was cut into pieces of 5 cm each and roasted, 1,650 g of mistletoe and 22,000 mL of water were added to the extractor, and the pressure was 0.15 kgf/cm^2^ at 110°C, and extraction was performed for 3 hours. After extraction, it was pasteurized for 40 minutes. The mistletoe extract was provided by sealing and refrigeration in a plastic pack of 110 mL (content of mistletoe, 13 g/pack).

Analysis by the Korea Advanced Food Research Institute (2016) found that the main chemical components of *mistletoe* extract were water (97%), crude ash (0.2%), crude fat (0.5%), crude protein (0.3%), carbohydrate (2.0%), sodium (3.9 mg/100 g), and flavonoids (32.0 mg/100 g).

### 2.4. Rowing Performance Test

An indoor rowing ergometer (Concept2 Inc., VT, USA) that is typically used for physical or technical training of rowing athletes was used to assess exercise performance. Performance in a 2,000 m rowing test was measured. This was a physically demanding task that required maximum effort from the subjects. During the rowing performance test, heart rate was maintained at ≥80% heart rate reserve, and changes in heart rate were measured every 5 s using a wireless heart rate meter (Polar RS400sd, Polar Electro Inc., NY, USA). Prior to the test, all subjects freely performed a warm-up on the rowing ergometer for 5 min.

### 2.5. Biochemical Analysis

Prior to blood collection, subjects were required to fast from 8:00 pm of the previous day. To measure cytokine levels at baseline, immediately after the 2,000 m rowing exercise, and following 30 min of recovery, 10 mL of blood was drawn from the forearm vein using a vacuum collection tube. The blood samples obtained were then centrifuged (Combi-514R, Hanil, Korea) for 10 min at 3,000 rpm. Serum was separated from blood cells, transferred to a microfuge tube, and was then stored at −80°C until analysis. Enzyme-linked immunosorbent assay (ELISA) was performed to determine serum concentration of IL-6 and TNF-*α* using CymaxTM Human IL-6 and TNF-*α* ELISA Kits (AB Frontier, Korea), respectively, and OD at 450 nm was measured using GENios (TECAN, Switzerland). An immunoturbidimetric assay with CRPL3 (Roche, Germany) and a Modular Analytics (Roche, Germany) analyzer was used to measure levels of CRP.

### 2.6. Statistical Analysis

Sample size was determined using a G-power version 3.1 Windows program (Kiel University, Kiel, Germany), based on a 0.25-point effect size (default), an alpha level of *p* < 0.05, and 60% power [30]. The mean value (*M*) and standard deviation (SD) of the measured parameters were calculated using the Statistical Package for the Social Sciences (SPSS) version 23.0 for Windows (SPSS Inc., Chicago, IL). To analyze interaction of differences between the groups (KME vs. CON, 2) and between periods (before vs. after, 2), a two-way repeated measures analysis of variance analysis was performed. To assess changes within each group before and after supplementation, a paired *t*-test was performed. An independent *t*-test was also used to analyze the delta (Δ) change. The level of statistical significance was set at *p* < 0.05.

## 3. Results

### 3.1. Subject Characteristics

The physical characteristics of study subjects are summarized in Table 1. There was no significant difference in mean age, height, weight, body mass index, and career achievements of the rowers in the KME and CON groups.

### 3.2. 2,000 m Rowing Ergometer Test

**Figure 2:**
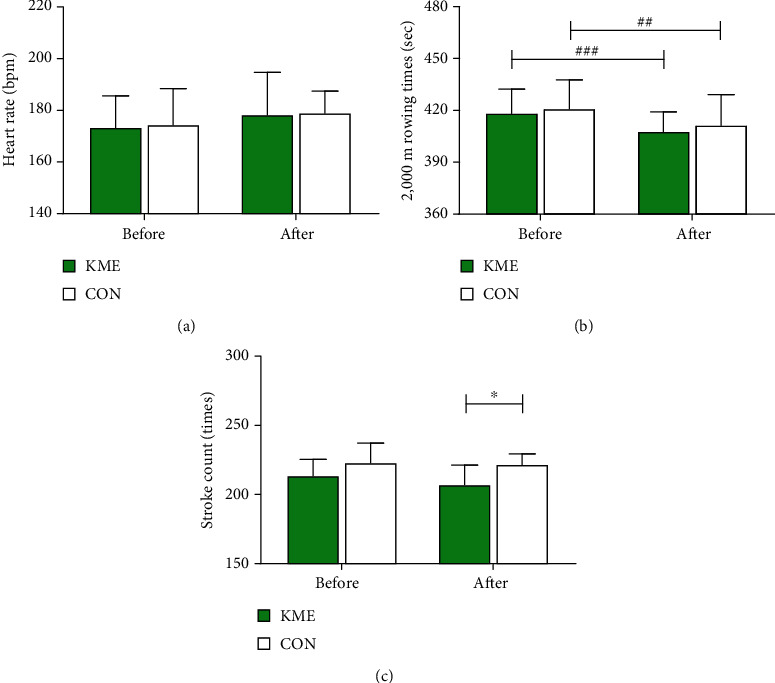
Before and after supplementation of 2,000 m rowing ergometer performance: (a) heart rate during the 2,000 m rowing test; (b) 2,000 m rowing time trial; (c) total stroke count during the 2,000 m rowing test. Data are shown as means ± SD. ^##^*p* < 0.01 and ^###^*p* < 0.001 before vs. after; ^∗^*p* < 0.05 KME vs. CON.

During the 2,000 m rowing ergometer trial test, there was no difference in heart rate between groups and periods (KME group before: 173.33 ± 12.26 bpm, after: 178.29 ± 16.43 bpm, *t* = −1.032, *p* = 0.329; CON group before: 174.39 ± 13.95 bpm, after: 178.91 ± 8.52 bpm, *t* = −1.514, *p* = 0.164). There was no significant difference in performance for the 2,000 m rowing test between the KME group (418.25 ± 14.03 s) and the CON group (420.98 ± 16.64 s) prior to the experiment (*t* = −.397, *p* = 0.696). The 2,000 m rowing test times significantly decreased in both the KME group (407.77 ± 11.39 s, *t* = 6.676, *p* < 0.001) and the CON group (411.33 ± 17.82 s, *t* = 3.770, *p* < 0.01) at after 8 weeks. Total rowing stroke count also did not differ significantly for the 2,000 m rowing test in both groups prior to supplementation. The KME group had a rowing stroke count of 213.60 ± 11.5, and the CON group had a count of 222.80 ± 14.40 (*t* = −1.566, *p* = 0.135). Following supplementation, total rowing stroke count was significantly different between the groups. The KME group (207.10 ± 14.11) had a lower rowing stroke count than the CON group (221.60 ± 7.82), as shown in Figure 2 (*t* = −2.843, *p* < 0.05).

### 3.3. Serum Inflammation Markers

#### 3.3.1. Interleukin-6 (IL-6)

**Figure 3:**
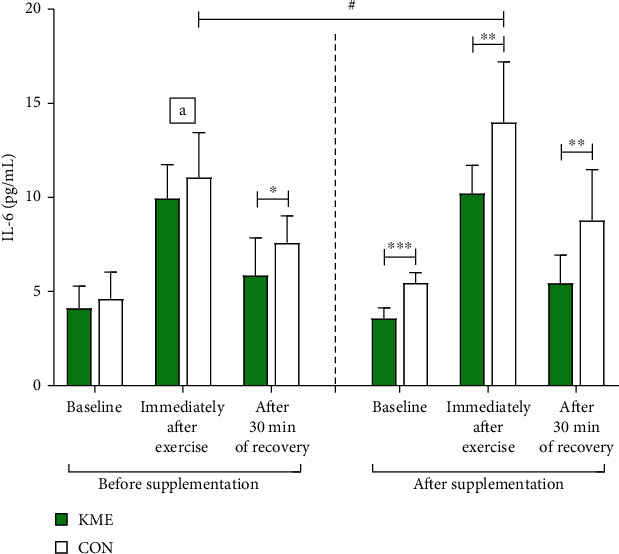
Changes in interleukin-6 levels during the 2,000 m rowing ergometer exercise trials performed before and after supplementation (means ± SD). ^a^Interaction effects immediately after exercise. ^#^*p* < 0.05 before vs. after; ^∗^*p* < 0.05, ^∗∗^*p* < 0.01, and ^∗∗∗^*p* < 0.001 KME vs. CON.

After the 8 weeks, baseline levels of serum IL-6 decreased from 4.13 ± 1.16 pg/mL to 3.57 ± 0.55 pg/mL in the KME group and increased from 4.62 ± 1.41 pg/mL to 5.46 ± 0.54 pg/mL in the CON group. The main effect of the two groups (*F* = 5.745, *p* < 0.05) after the 8 weeks was different at baseline (*t* = −7.760, *p* < 0.001). Immediately following exercise, the concentration of serum IL-6 in the KME group was 9.95 ± 1.79 pg/mL before and 10.22 ± 1.49 pg/mL after supplementation. In the CON group, the concentration of serum IL-6 increased from 11.07 ± 2.38 pg/mL to 13.99 ± 3.21 pg/mL after the 8 weeks. An interaction effect (*F* = 6.596, *p* < 0.05) and the main effect of the groups (*F* = 12.117, *p* < 0.01) were observed immediately following exercise. Serum IL-6 significantly increased immediately after exercise in the CON group (*t* = −2.443, *p* < 0.05). There were significant differences between the groups after the 8 weeks (*t* = −3.373, *p* < 0.01). The concentration of serum IL-6 in the KME group after 30 min of recovery was 5.86 ± 1.98 pg/mL before supplementation and 5.45 ± 1.49 pg/mL after supplementation. In the CON group, the concentration of serum IL-6 was 7.59 ± 1.43 pg/mL before and 8.78 ± 2.70 pg/mL after the 8 weeks. The main effect of the groups (*F* = 7.748, *p* < 0.05) and comparison of differences between the two groups revealed that they were significantly different before (*F* = −2.229, *p* < 0.05) and after the 8-week period (*F* = −3.425, *p* < 0.01), as seen in Figure 3.

**Figure 4:**
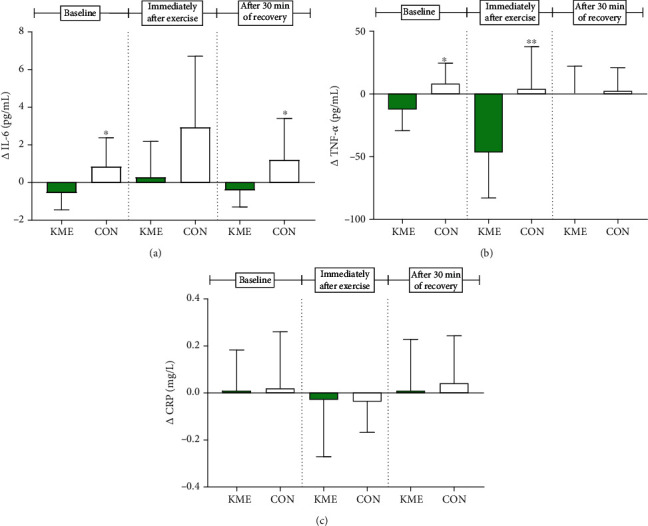
Change in inflammation biomarkers. (a) Significant differences were observed at baseline and after 30 min of recovery in the delta (Δ) values of IL-6 (^∗^*p* < 0.05, ^∗^*p* < 0.05). (b) Significant differences were observed at baseline and immediately after exercise in the delta (Δ) values of TNF-*α* (^∗^*p* < 0.05, ^∗∗^*p* < 0.01). (c) Δ values of CRP, no significant differences were observed. Data are shown as means ± SD.

Differences (Δ) between serum levels of IL-6 before and after the 8 weeks are represented in Figure 4(a). Briefly, the baseline of Δ-IL-6 was −0.56 ± 0.90 pg/mL in the KME group and 0.84 ± 1.54 pg/mL in the CON group. This was significantly different (*t* = −2.493, *p* < 0.05). Immediately following exercise, the Δ-IL-6 was 0.27 ± 1.91 pg/mL in the KME group and 2.92 ± 3.79 pg/mL in the CON group (*t* = −1.978, *p* = 0.063). After 30 min of recovery, the Δ-IL-6 was −0.42 ± 0.88 pg/mL in the KME group and 1.19 ± 2.22 pg/mL in the CON group. This was again significantly different (*t* = −2.136, *p* < 0.05).

#### 3.3.2. Tumor Necrosis Factor-Alpha (TNF-*α*)

**Figure 5:**
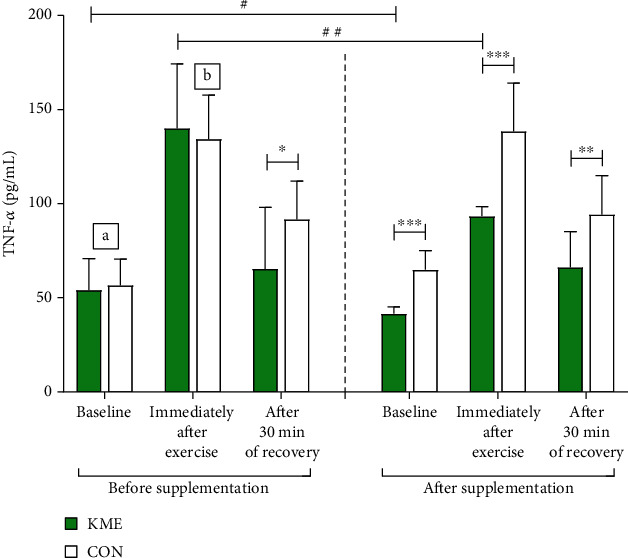
Changes in tumor necrosis factor alpha levels during the 2,000 m rowing ergometer exercise trials before and after supplementation (means ± SD). ^a^Interaction effects at the baseline level. ^b^Interaction effects immediately after exercise. ^#^*p* < 0.05 and ^##^*p* < 0.01 before vs. after; ^∗^*p* < 0.05, ^∗∗^*p* < 0.01, and ^∗∗∗^*p* < 0.001 KME vs. CON.

An interaction effect (*F* = 8.678, *p* < 0.05) was observed for baseline levels of serum TNF-*α*; there was a significant main effect of group (*F* = 7.612, *p* < 0.05). Concentration of serum TNF-*α* decreased from 54.05 ± 16.75 pg/mL to 41.51 ± 3.68 pg/mL after supplementation in the KME group. In contrast, in the CON group, concentration of serum TNF-*α* was 56.57 ± 13.90 pg/mL prior to and 64.89 ± 10.06 after the 8 weeks. This demonstrated that the KME group had significantly lower TNF-*α* levels (*t* = 2.360, *p* < 0.05). Significant differences were observed between the two groups after the 8 weeks (*F* = −6.900, *p* < 0.001). Immediately following exercise, concentration of serum TNF-*α* in the KME group decreased from 140.04 ± 34.17 pg/mL to 93.27 ± 5.10 pg/mL after supplementation. In the CON group, concentration of serum TNF-*α* was 134.28 ± 23.27 pg/mL before and 138.46 ± 25.59 pg/mL after the 8 weeks. There was an interaction effect (*F* = 8.201, *p* < 0.05), a main effect of group (*F* = 6.169, *p* < 0.05), and main effect of time (*F* = 10.827, *p* < 0.01). The decrease observed in the KME group was statistically significant (*t* = 4.092, *p* < 0.01). After the 8-week period, the difference between the two groups was also statistically significant (*t* = −5.477, *p* < 0.001). After 30 min of recovery, serum concentration of TNF-*α* in the KME group was 65.31 ± 32.71 pg/mL prior to supplementation and 66.14 ± 19.05 pg/mL after supplementation. In the CON group, serum concentration of TNF-*α* was 91.68 ± 20.32 pg/mL prior to and 94.22 ± 20.64 pg/mL after the 8 weeks. There was no statistically significant interaction. We observed significant differences between the two groups prior to (*t* = −2.165, *p* < 0.05) and after supplementation (*t* = −3.161, *p* < 0.01), as shown in Figure 5.

Differences (Δ) in levels of TNF-*α* before and after the 8-week period are presented in Figure 4(b). The baseline Δ-TNF-*α* was −12.54 ± 16.81 pg/mL in the KME group and 8.32 ± 16.23 pg/mL in the CON group. This difference was significant (*t* = −2.823, *p* < 0.05). Immediately following exercise, Δ-TNF-*α* was −46.77 ± 36.14 in the KME group and 4.18 ± 33.38 pg/mL in the CON group (*t* = −3.275, *p* < 0.01). After 30 min of recovery, Δ-TNF-*α* was 0.83 ± 21.26 pg/mL in the KME group and 2.54 ± 18.43 in the CON group (*t* = −0.192, *p* = 0.850).

#### 3.3.3. C-Reactive Protein (CRP)

**Figure 6:**
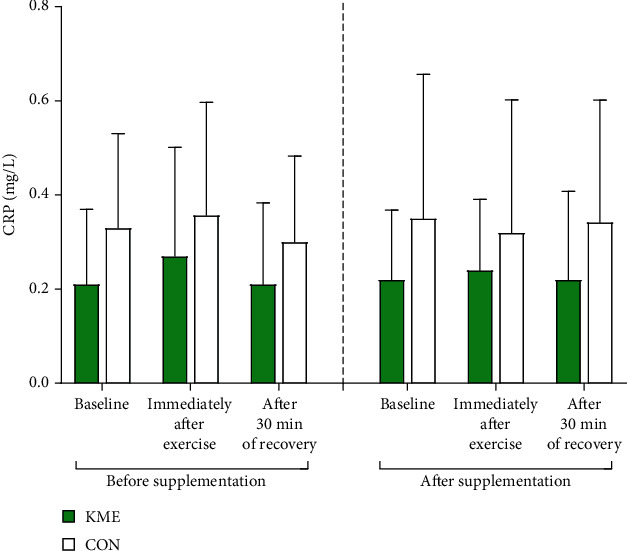
Changes in C-reactive protein levels during the 2,000 m rowing ergometer exercise trials before and after supplementation (means ± SD). There was no difference.

Baseline concentration of serum CRP in the KME group was 0.21 ± 0.16 mg/L prior to and 0.22 ± 0.15 mg/L after supplementation. In the CON group, it was 0.33 ± 0.20 mg/L before and 0.35 ± 0.31 mg/L after the 8-week period. Immediately following exercise, concentration of serum CRP in the KME group was 0.27 ± 0.23 mg/L prior to and 0.24 ± 0.15 mg/L after supplementation. In the CON group, it was 0.36 ± 0.24 mg/L before and 0.32 ± 0.28 after the 8-week period. After 30 min of recovery, the concentration of serum CRP in the KME group was 0.21 ± 0.17 mg/L prior to and 0.22 ± 0.19 mg/L after supplementation. In the CON group, the concentration was 0.30 ± 0.18 mg/L before and 0.34 ± 0.26 mg/L after the 8-week period. No interaction effect was observed, and there were no significant differences between the two groups (Figure 6).

Differences (Δ) in concentration of serum CRP before and after the 8-week period are represented in Figure 4(c). There were no significant differences observed for all time points. The baseline for Δ-CRP in the KME group was 0.01 ± 0.17 mg/L and was 0.02 ± 0.24 mg/L in the CON group (*t* = −0.107, *p* = 0.916). Immediately following exercise, Δ-CRP was −0.03 ± 30.24 mg/L in the KME group and −0.04 ± 0.13 mg/L in the CON group (*t* = 0.081, *p* = 0.936). After 30 min of recovery, Δ-CRP was 0.01 ± 0.22 mg/L in the KME group and 0.04 ± 0.21 mg/L in the CON group (*t* = −0.341, *p* = 0.737).

## 4. Discussion

This study demonstrated the effect of *mistletoe* supplementation on the release of proinflammatory cytokines induced by strenuous exercise. Immediately following a bout of strenuous exercise (a 2,000 m rowing ergometer trial), there was a rapid increase in serum inflammatory cytokines such as IL-6 and TNF-*α*. Supplementation with *mistletoe* extract after exercise for the purposes of quick recovery dampened the increase in IL-6 and TNF-*α* from baseline levels. Daily administration of the *mistletoe* extract supplement for 8 weeks prevented possible increases in the levels of IL-6 and TNF-*α* induced by long training periods. Immediately following a break after strenuous exercise, levels of serum IL-6 and TNF-*α* decreased. Our data suggest that *mistletoe* extract decreases levels of inflammation in elite athletes. This is the first study to investigate the effects of such an antioxidant treatment on exercise-induced oxidative stress.

Training protocols include long training times and high-intensity training for the purposes of reinforcing skills and improving performance. In this study, the subjects performed high-intensity training for 8 weeks. During the off-season period, performance of rowing athletes in a 2,000 m rowing ergometer trial was compared pre- and postsupplementation with *mistletoe* extract. Our results demonstrated that the time taken to row 2,000 m on the rowing ergometer significantly decreased in each group. As both groups had undertaken 8 weeks of training, the time for the trial decreased compared to the baseline level in both groups. In particular, the KME group reduced the record of 2,000 m rowing performance. Furthermore, the total number of strokes was significantly lower in the KME group compared to the CON group.

*Mistletoe* extract stimulates the release of peroxisome proliferator-activated receptor gamma coactivator 1-alpha and sirtuin 1 in myoblasts, resulting in improved endurance by upregulating mRNA expression of genes associated with mitochondrial creation and function [24]. It has also been shown to increase expression of mitochondrial uncoupling protein-3 in a myoblast cell line, which activates adenosine monophosphate-activated protein kinase (AMPK) [31]. Another animal study on *mistletoe* extract supplement enhancement of endurance reported that administration of *mistletoe* extract for 12 weeks extended time to exhaustion on the treadmill by 2.5 times and swimming time by 212% and reduced postexercise lactate levels in the experimental group compared to the control group [24]. Administration of *mistletoe* extract during treadmill exercise also increased distance and duration of exercise till exhaustion compared to control [31]. This suggests that rowing performance improvements in the KME group that was administered *mistletoe* extract may be due to increased capacity in the electron transport chain system through increased oxygen consumption by mitochondria [24]. Alternatively, improvements may be due to changes in muscle function and muscle differentiation index [32].

Several mechanisms are involved in inflammatory responses: (1) detection of harmful stimuli *via* binding to the cell surface receptor, (2) revitalizing inflammatory channels, (3) release of inflammatory molecules, and (4) recruitment of inflammatory cells [12]. Cytokines regulate inflammation through a complex network of interactions and calibrate immune response to infection or inflammation. However, excessive inflammatory cytokine production can cause damage including tissue injury, hemodynamic changes, and organ insufficiency that ultimately results in death [33, 34]. An appropriate level of IL-6 maintains glucose homeostasis in muscle tissue and activates the production of AMPK in skeletal muscles to stimulate absorption of glucose and fat oxidation [35]. IL-6 plays a major role in anti-inflammation by blocking the IL-1 receptor and directly inhibiting the expression of TNF-*α* and IL-1*β* and is therefore classified as both a pro- and anti-inflammatory cytokine [36]. However, a rapid increase in IL-6 causes cytokine imbalance and induces a critical inflammation state. Athletes who engage in intensive strength-based exercise for long periods are vulnerable to chronic inflammatory conditions [14], and this form of physical exertion results in increased levels of inflammatory cytokines in the blood [37]. Inflammation is accompanied by muscle pain and discomfort, which increases risk of injury and reduces performance of athletes [38].

Korean *mistletoe* has higher inhibition of lipid peroxidation than an equivalent concentration (10 mg) of vitamin C and demonstrates high antioxidative properties [39]. Oak tree *mistletoe* plays a role as a natural antioxidant as it contains polyphenols, has a superior ability to donate electrons, and increases activity of superoxide dismutase [40]. In particular, homoflavoyadorinin-B, a bioactive flavonoid found exclusively in *mistletoe*, shows potential in preventing and treating diseases associated with the generation of reactive oxygen species [41]. *Mistletoe*'s anti-inflammatory effects also include inhibition of cytokines induced by prostaglandin E2 biosynthesis through selective inhibition of cyclooxygenase-2 (COX-2) [20]. It has also recently been suggested that Korean *mistletoe* may exert anti-inflammatory effects *via* activating transcription factor 3 and mitogen-activated protein kinase (MAPK) and inhibiting NF-*κ*B signal transduction, decreasing inflammatory effectors such as inducible nitric oxide synthases (iNOS), COX-2, TNF-*α*, and interleukin-1 beta (IL-1*β*) [42].

Serum TNF-*α* was elevated after the 2,000 m rowing trial and induced IL-6 expression. The KME group had a lower concentration of IL-6 and TNF-*α* compared to the CON group after 30 min of recovery, both before and after postexercise supplementation. This indicated that supplementation with *mistletoe* extract was effective in treating acute exercise-induced inflammation when coupled with 30 min of recovery. The KME group also had a lower concentration of serum inflammatory cytokines compared to the CON group at baseline, immediately after exercise, and after 30 min of recovery postsupplementation. This finding implies that supplementation with *mistletoe* ameliorates inflammatory responses induced by long-term intensive training.

However, despite the rapid increase in serum inflammatory cytokines after the 2,000 m rowing trial, there was no significant change in the level of CRP and no interaction effect. Acute phase proteins including CRP also increased after excessive exercise, but it was confirmed that the increase was delayed compared to IL-6 and TNF-*α*. Because CRP increases dramatically during the inflammatory process and remains elevated for long periods of time, it is thought to be considered in future studies of chronic inflammation levels in athletes.

## 5. Conclusions

Supplementation with *mistletoe* extract had anti-inflammatory and positive effects on performance during long-term high-intensity training and extreme exertion (a 2,000 m rowing trial). Thus, we propose that *mistletoe* extract may be a valuable anti-inflammatory supplement that protects against exercise-induced oxidative damage in athletes who have increased inflammation due to high-intensity training.

## Figures and Tables

**Table 1:** Physical characteristics of subjects in both groups.

Variables	Age (yr)	Height (cm)	Weight (kg)	BMI (kg/m^2^)	Career (yr)
Group					
KME (*n* = 10)	20.44 ± 0.99	180.87 ± 5.03	81.73 ± 8.52	24.99 ± 1.56	5.70 ± 1.34
CON (*n* = 10)	20.30 ± 1.42	179.05 ± 3.53	79.41 ± 9.24	24.76 ± 3.03	5.20 ± 1.55

Values are means ± SD. KME: Korean mistletoe extract intake group; CON: control group.

## Data Availability

The data used to support the findings of this study are available from the corresponding author upon request.

## Abbreviations

KME: Korean *mistletoe* extract
IL-6: Interleukin-6
TNF-*α*: Tumor necrosis factor-alpha
CRP: C-reactive protein
NF-*κ*B: Nuclear factor kappa-light-chain-enhancer of activated B cells
COX-2: Cyclooxygenase-2
MAPK: Mitogen-activated protein kinase
iNOS: Inducible nitric oxide synthases
IL-1*β*: Interleukin-1 beta.

## Additional Points

*Highlights*. (i) Korean *mistletoe* extract (KME) has shown functional foods for anti-inflammation. (ii) Inflammatory cytokine expression increased dramatically after strenuous exercise. (iii) KME supplementation prevented increases in IL-6 and TNF-*α* levels induced by high-intensity training. (iv) KME was consequently helpful in recovery.

## References

[1] da Rocha A. L., Pinto A. P., Kohama E. B. (2019). The proinflammatory effects of chronic excessive exercise. *Cytokine*.

[2] Mooren F. C., Lechtermann A., Volker K. (2004). Exercise-induced apoptosis of lymphocytes depends on training status. *Medicine and Science in Sports and Exercise*.

[3] Fontes F. L., Pinheiro D. M., Oliveira A. H., Oliveira R. K., Lajus T. B., Agnez-Lima L. F. (2015). Role of DNA repair in host immune response and inflammation. *Mutation Research, Reviews in Mutation Research*.

[4] de la Fuente H., Cibrian D., Sanchez-Madrid F. (2012). Immunoregulatory molecules are master regulators of inflammation during the immune response. *FEBS Letters*.

[5] Smith L. L. (2000). Cytokine hypothesis of overtraining: a physiological adaptation to excessive stress?. *Medicine and Science in Sports and Exercise*.

[6] Vargas N. T., Marino F. (2014). A neuroinflammatory model for acute fatigue during exercise. *Sports Medicine*.

[7] Macedo Santiago L. A., Neto L. G. L., Borges Pereira G. (2018). Effects of resistance training on immunoinflammatory response, TNF-alpha gene expression, and body composition in elderly women. *Journal of Aging Research*.

[8] Vallabhapurapu S., Karin M. (2009). Regulation and function of NF-*κ*B transcription factors in the immune system. *Annual Review of Immunology*.

[9] Glund S., Deshmukh A., Long Y. C. (2007). Interleukin-6 directly increases glucose metabolism in resting human skeletal muscle. *Diabetes*.

[10] Elenkov I. J., Chrousos G. P., Wilder R. L. (2000). Neuroendocrine regulation of IL-12 and TNF-alpha/IL-10 balance. Clinical implications. *Annals of the New York Academy of Sciences*.

[11] Petersen A. M., Pedersen B. K. (2005). The anti-inflammatory effect of exercise. *Journal of Applied Physiology (Bethesda, MD: 1985)*.

[12] Chen L., Deng H., Cui H. (2018). Inflammatory responses and inflammation-associated diseases in organs. *Oncotarget*.

[13] Bermon S., Castell L. M., Calder P. C. (2017). Consensus statement immunonutrition and exercise. *Exercise Immunology Review*.

[14] Scherr J., Nieman D. C., Schuster T. (2012). Nonalcoholic beer reduces inflammation and incidence of respiratory tract illness. *Medicine and Science in Sports and Exercise*.

[15] Lima L. C. R., Assumpcao C. O., Prestes J., Denadai B. S. (2015). Consumption of cherries as a strategy to attenuate exercise-induced muscle damage and inflammation in humans. *Nutrición Hospitalaria*.

[16] Myburgh K. H. (2014). Polyphenol supplementation: benefits for exercise performance or oxidative stress?. *Sports Medicine*.

[17] Funes L., Carrera-Quintanar L., Cerdan-Calero M. (2011). Effect of lemon verbena supplementation on muscular damage markers, proinflammatory cytokines release and neutrophils’ oxidative stress in chronic exercise. *European Journal of Applied Physiology*.

[18] Szurpnicka A., Zjawiony J. K., Szterk A. (2019). Therapeutic potential of mistletoe in CNS-related neurological disorders and the chemical composition of Viscum species. *Journal of Ethnopharmacology*.

[19] Kienle G. S., Kiene H. (2007). Complementary cancer therapy: a systematic review of prospective clinical trials on anthroposophic mistletoe extracts. *European Journal of Medical Research*.

[20] Hegde P., Maddur M. S., Friboulet A., Bayry J., Kaveri S. V. (2011). *Viscum album* exerts anti-inflammatory effect by selectively inhibiting cytokine-induced expression of cyclooxygenase-2. *PLoS One*.

[21] Fidan I., Ozkan S., Gurbuz I. (2008). The efficiency of *Viscum album* ssp. album and Hypericum perforatum on human immune cells in vitro. *Immunopharmacology and Immunotoxicology*.

[22] Ojewole J. A., Adewole S. O. (2007). Hypoglycaemic and hypotensive effects of Globimetula cupulata (DC) Van Tieghem (Loranthaceae) aqueous leaf extract in rats. *Cardiovascular Journal of South Africa*.

[23] Kim B. K., Choi M. J., Park K. Y., Cho E. J. (2010). Protective effects of Korean mistletoe lectin on radical-induced oxidative stress. *Biological & Pharmaceutical Bulletin*.

[24] Jung H. Y., Lee A. N., Song T. J. (2012). Korean mistletoe (*Viscum album* coloratum) extract improves endurance capacity in mice by stimulating mitochondrial activity. *Journal of Medicinal Food*.

[25] Kim K. W., Yang S. H., Kim J. B. (2014). Protein fractions from Korean mistletoe (*Viscum album* coloratum) extract induce insulin secretion from pancreatic beta cells. *Evidence-based Complementary and Alternative Medicine*.

[26] Kim J. H., Kim D. Y., Lee J. A., Ha H. D. (2015). Effects of aquatic dynamic recovery on blood lactate, ammonia, LDH and CK after performing rowing ergometer in rowers. *Journal of Coach Develop*.

[27] Lee Y. H., Shin K. O., Kim K. S., Kim Y. I., Woo J. H. (2011). The effects of D-ribose supplementation on the production of blood fatigue factors after maximal intensity exercise. *Journal of Life Science*.

[28] Ha M. S., Kim J. H., Ha S. M., Kim Y. S., Kim D. Y. (2019). Positive influence of aqua exercise and burdock extract intake on fitness factors and vascular regulation substances in elderly. *Journal of Clinical Biochemistry and Nutrition*.

[29] Kwak J. J., Yook J. S., Ha M. S. (2020). Potential biomarkers of peripheral and central fatigue in high-intensity trained athletes at high-temperature: a pilot study with *Momordica charantia* (bitter melon). *Journal of Immunology Research*.

[30] Faul F., Erdfelder E., Lang A. G., Buchner A. (2007). G∗Power 3: a flexible statistical power analysis program for the social, behavioral, and biomedical sciences. *Behavior Research Methods*.

[31] Lee S.-H., Kim I.-B., Kim J.-B., Park D.-H., Min K.-J. (2014). The effects of Korean mistletoe extract on endurance during exercise in mice. *Animal Cells and Systems*.

[32] Lim N. J., Shin J. H., Kim H. J. (2017). A combination of Korean mistletoe extract and resistance exercise retarded the decline in muscle mass and strength in the elderly: a randomized controlled trial. *Experimental Gerontology*.

[33] Czaja A. J. (2014). Review article: chemokines as orchestrators of autoimmune hepatitis and potential therapeutic targets. *Alimentary Pharmacology & Therapeutics*.

[34] Liu Z., Wang Y., Wang Y. (2016). Dexmedetomidine attenuates inflammatory reaction in the lung tissues of septic mice by activating cholinergic anti-inflammatory pathway. *International Immunopharmacology*.

[35] Kahn B. B., Alquier T., Carling D., Hardie D. G. (2005). AMP-activated protein kinase: ancient energy gauge provides clues to modern understanding of metabolism. *Cell Metabolism*.

[36] Pedersen B. K., Steensberg A., Schjerling P. (2001). Exercise and interleukin-6. *Current Opinion in Hematology*.

[37] Bruunsgaard H. (2005). Physical activity and modulation of systemic low-level inflammation. *Journal of Leukocyte Biology*.

[38] Moreira A., Kekkonen R. A., Delgado L., Fonseca J., Korpela R., Haahtela T. (2007). Nutritional modulation of exercise-induced immunodepression in athletes: a systematic review and meta-analysis. *European Journal of Clinical Nutrition*.

[39] Song H.-S., Park Y.-H., Kim S.-K., Moon W.-K., Kim D.-W., Moon K.-Y. (2004). Downregulatory effect of extracts from mistletoe (*Viscum album*) and pueraria root (*Pueraria radix*) on cellular NF-*κ*B activation and heir antioxidant activity. *Journal of the Korean Society of Food Science and Nutrition*.

[40] Lee H.-J., Do J.-R., Kwon J.-H., Kim H.-K. (2011). Physiological properties of oak mistletoe (*Loranthus yadoriki*) extracts by microwave extraction condition. *Korean Journal of Food Preservation*.

[41] Choi S.-Y., Chung S.-K., Kim S.-K. (2004). An antioxidant homo-flavoyadorinin-B from Korean mistletoe (*Viscum album* var. coloratum). *Journal of Applied Biological Chemistry*.

[42] Park S. B., Park G. H., Kim H. N. (2018). Anti-inflammatory effect of the extracts from the branch of *Taxillus yadoriki* being parasitic in *Neolitsea sericea* in LPS-stimulated RAW264.7 cells. *Biomedicine & Pharmacotherapy*.

